# Suppressing synchronous firing of epileptiform activity by high‐frequency stimulation of afferent fibers in rat hippocampus

**DOI:** 10.1111/cns.13535

**Published:** 2020-12-16

**Authors:** Zhaoxiang Wang, Zhouyan Feng, Yue Yuan, Lvpiao Zheng

**Affiliations:** ^1^ Key Laboratory for Biomedical Engineering of Ministry of Education College of Biomedical Engineering & Instrument Science Zhejiang University Hangzhou China

**Keywords:** afterdischarge, burst, de‐synchronization, epilepsy, high‐frequency stimulation, hippocampal CA1 region

## Abstract

**Aims:**

Deep brain stimulation (DBS) is a promising technology for treating epilepsy. However, the efficacy and underlying mechanisms of the high‐frequency stimulation (HFS) utilized by DBS to suppress epilepsy remain uncertain. Previous studies have shown that HFS can desynchronize the firing of neurons. In this study, we investigated whether the desynchronization effects of HFS can suppress epileptiform events.

**Methods:**

HFS trains with seconds of duration (short) and a minute of duration (long) were applied at the afferent fibers (ie, Schaffer collaterals) of the hippocampal CA1 region in anesthetized rats in vivo. The amplitude and the rate of population spikes (PS) appeared in the downstream of stimulation were calculated to evaluate the intensity of synchronized firing of neuronal populations between short and long HFS groups. A test of paired‐pulse depression (PPD) was used to assess the alteration of inhibitory neuronal circuits.

**Results:**

The sustained stimulation of a 60‐s long HFS suppressed the afterdischarges that were induced by a 5‐s short HFS to impair the local inhibitions. During the sustained HFS, the mean PS amplitude reduced significantly and the burst firing decreased, while the amount of neuronal firing did not change significantly. The paired‐pulse tests showed that with a similar baseline level of small PS2/PS1 ratio indicating a strong PPD, the 5‐s HFS increased the PS2/PS1 ratio to a value that was significantly greater than the corresponding ratio during sustained HFS, indicating that the PPD impaired by a short HFS may be restored by a sustained HFS.

**Conclusions:**

The sustained HFS can desynchronize the population firing of epileptiform activity and accelerate a recovery of inhibitions to create a balance between the excitation and the inhibition of local neuronal circuits. The study provides new clues for further understanding the mechanism of DBS and for advancing the clinical application of DBS in treating epilepsy.

## Graphical abstract

Sustained high‐frequency stimulations (HFS) of 100 Hz pulses can suppress the population spikes (PS) in the epileptic afterdischarges induced by a short HFS in the rat hippocampal region. The possible mechanisms are a desynchronization of neuronal firing and a recovery of inhibitions in the local neuronal circuits. The findings provide new clues for developing brain stimulation therapy.

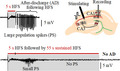

## 1 INTRODUCTION

Deep brain stimulation (DBS) has emerged in the past decades as a promising therapy for neurological and psychiatric disorders, including Parkinson's disease, epilepsy, depression, and obsessive compulsive disorder.^1^, ^2^ However, so far the most successful application of DBS is still limited in treating motor disorders of Parkinson's disease.^3^, ^4^ Although studies of electrical stimulations in brain to control epilepsy can be traced back to the 1950s,^5^ DBS therapy for treating epilepsy has not been applied widely because its efficacy and effects on epilepsy are still uncertain.^4^ Considering the high incidence of epilepsy in human beings, especially the high proportion of refractory epilepsy, it is significant to promote the DBS for treating epilepsy.

Currently, DBS usually utilizes high‐frequency stimulation (HFS) of pulse trains with a frequency over 90 Hz to control epilepsy.^4^, ^6^ Studies have shown that HFS trains can decrease the neuronal firing in the site close to the stimulating electrode. Possible mechanisms of the suppression include the followings: activating inhibitory synapses,^7^ decreasing inputs of excitatory synapses,^8^, ^9^ and directly exciting the soma membrane excessively to a level of depolarization block thereby preventing its generation of action potentials.^10^, ^11^ However, even if the soma of a neuron at the site of stimulation is suppressed, its axon can still generate action potentials under the direct impulse of HFS.^7^, ^12^, ^13^ This may be a reason why many of animal and clinical studies have shown that HFS can increase epileptiform activity.^14^, ^15^, ^16^ The axon of a neuron is more prone to be activated by extracellular pulses of HFS than the other structures of a neuron, such as the soma and dendrites.^17^, ^18^ Action potential generated at the axon can propagate along the axon to the postsynaptic neurons, thereby exciting the downstream brain regions.^12^, ^19^ The excitatory effect of HFS on axons may promote certain types of epilepsy. For instance, HFS may induce epileptiform activity through an effect of "kindling".^20^, ^21^

Nevertheless, recent animal studies have shown that the neuronal firing induced by prolonged HFS in the downstream region of stimulation is asynchronous.^22^, ^23^ Moreover, computational studies have suggested that the HFS with commonly used pulse frequencies (83 ‐ 200 Hz) may have a crucial effect that is to desynchronize the firing of neuronal populations rather than to decrease the firing rates of individual neurons.^24^, ^25^ Because abnormal increases of both the firing rates and the firing synchronization of neuronal populations are the two major causes of epilepsy generations,^26^, ^27^ a decrease of firing amount or/and a decrease of firing synchronization may suppress epileptiform activity.^28^, ^29^ In this context, we hypothesize that the HFS could suppress epileptiform activity by decreasing the firing synchronization of neuronal populations.

To test this hypothesis, we applied HFS on the afferent fibers (ie, the Schaffer collateral) of the hippocampal CA1 region in anaesthetized rats to evaluate the effect of sustained stimulation of a long HFS on afterdischarges (AD) induced by a short HFS. Since the hippocampal region is one of the major focuses of epilepsy,^4^, ^30^ the results of this study could reveal new mechanism of DBS for treating epilepsy and provide clues for developing new stimulation paradigms of DBS.

## 2 MATERIALS AND METHODS

### 2.1 Surgery and electrode implantation

All surgical procedures used in this study were in accordance with the Guide for the Care and Use of Laboratory Animals (China Ministry of Health). The protocol was approved by the Institutional Animal Care and Use Committee, Zhejiang University. Forty‐five adult male Sprague‐Dawley rats (320 ± 41 g) were used under anesthesia with urethane (1.25g/kg, i.p.). The anesthetized rats were placed in a stereotaxic apparatus (Stoelting Co.) during experiments.

**Figure 1:**
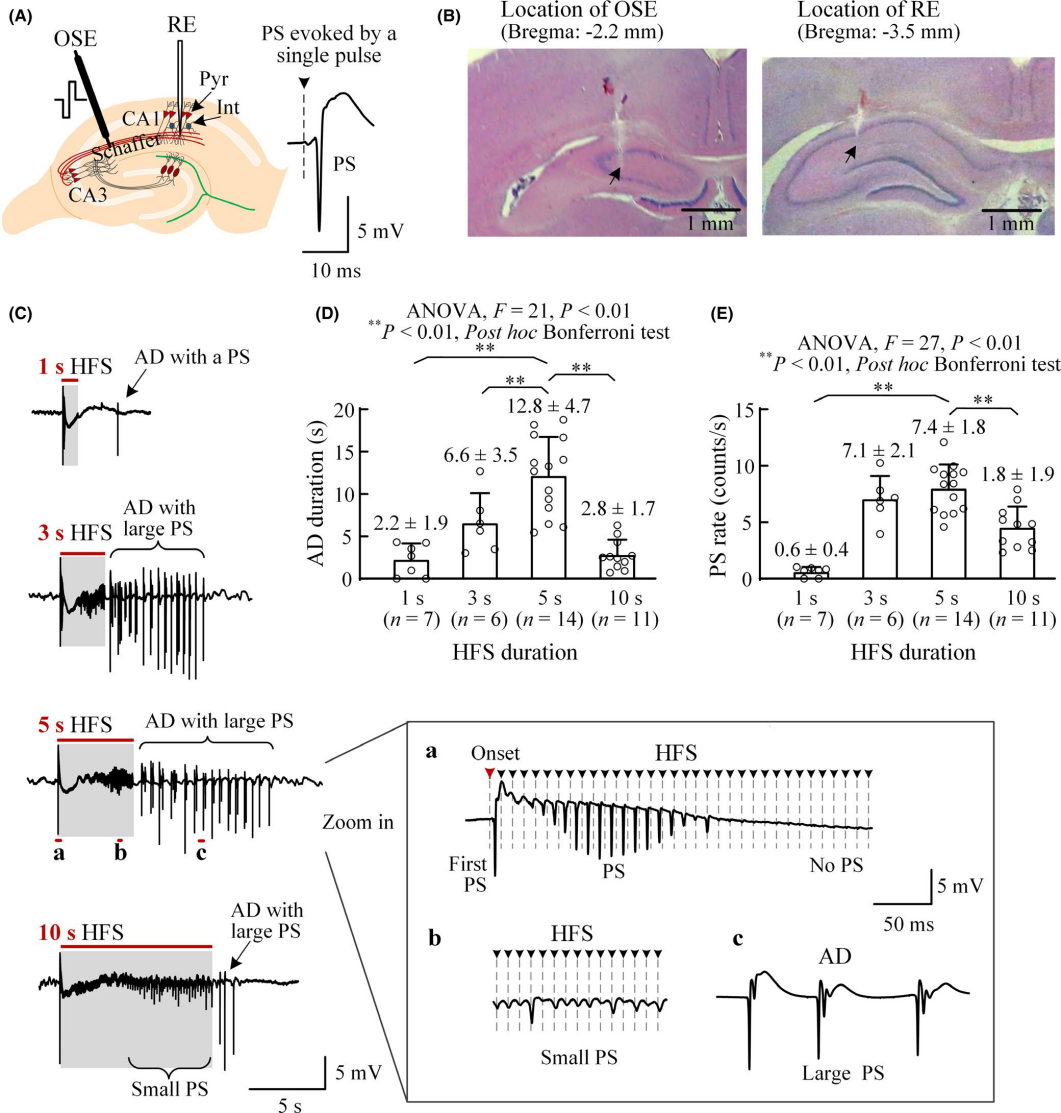
Afterdischarges (AD) induced by short HFS trains in rat hippocampal CA1 region in vivo. (A) Schematic diagram of the locations of the orthodromic‐stimulation electrode (OSE) at Schaffer collaterals and the recording electrode (RE) in the CA1 region. A typical population spike (PS) orthodromically evoked by a single pulse is shown on the right. (B) Photographs of histological brain slices demonstrating the electrode tracks of OSE and RE in the coronal sections of ~ 2.2 and ~ 3.5 mm posterior to bregma, respectively. (C) Typical examples of afterdischarges with large PS events induced by HFS trains with a duration of 1, 3, 5, and 10 s, respectively. The red bars and shades denote HFS durations. The expanded insets at the right show typical PS waveforms in different periods. (D) and (E) Comparisons of the AD durations and PS rates induced by HFS with 1, 3, 5, and 10 s durations. In A and C (insets), arrows with dashed lines denote the removed artifacts of stimulation pulses

The recording electrode, a 16‐channel electrode array with each contact area of 177 μm^2^ (Model Poly2, NeuroNexus Technologies Inc, USA), was inserted in the hippocampal CA1 region (AP −3.5; ML 2.7; DV 2.5). The stimulating electrode, a concentric bipolar electrode (Model CBCSG75, FHC Inc, USA), was inserted in the Schaffer collaterals of CA1 region (AP −2.2; ML 2.0; DV 2.8) to orthodromically activate the neuronal populations in the recording site, that is, the downstream of the stimulation (Figure 1A). The waveforms of both spontaneous unit spikes and evoked potentials recorded in the CA1 region along the array were used to guide the final locations of the two electrodes. The details of animal surgery and electrode placements were similar to the previous reports.^22^, ^31^

After experiment, the anesthetized rat was transcardially perfused with phosphate buffer saline (PBS) followed by 4% paraformaldehyde for ~ 40 minutes. Its brain was then isolated and put into 4% paraformaldehyde overnight at 4°C. Brain slices were obtained by using paraffin section with 4 µm serial sections. Hematoxylin‐eosin staining was applied on the slices to confirm the locations of the stimulating and recording electrodes (Figure 1B).

### 2.2 Recording and stimulating

The electrical signals collected by the recording array were amplified 100 times by a 16‐channel amplifier (Model 3600, A‐M Systems Inc, USA) with a band‐pass filtering range of 0.3‐5000 Hz. The amplified signals were then sampled by a PowerLab data acquisition system (Model PL3516, ADInstruments Inc, Australia) with a sampling rate of 20 kHz.

The stimuli, HFS trains of biphasic current pulses with each phase width of 0.1 ms, were generated by a programmable stimulator (Model 3800, A‐M Systems Inc, USA). The pulse frequency was 100 Hz. The pulse intensity was 0.3 ‐ 0.4 mA which was able to evoke a population spike (PS) with approximately 3/4 maximal amplitude in the pyramidal layer of CA1 region. The duration of a short HFS train was 1, 3, 5, or 10 s, and the duration of a long HFS train was 60 s.

To avoid a possible interference of long‐term potentiation (LTP) induced by a short HFS train on subsequent experimental outcomes, the short HFS trains with different durations were carried out in different rat preparations. In some experiments, a 60‐s long HFS train was paired with a 5‐s short HFS train in the same preparation. Previous studies have shown that the activity of neurons can recover to their baseline levels several minutes following the end of 60‐s HFS.^31^, ^32^ Therefore, in these paired stimulations, a 60‐s HFS was applied first and then followed by a 5‐s HFS after an interval of ~ 30 minutes to avoid possible interferences from the previous HFS train.

To simplify descriptions, for a 60‐s long HFS, the remaining stimulation period following the initial 5‐s was termed as sustained HFS.

### 2.3 Data analysis

Due to the heavy density of neurons in hippocampal CA1 region, the synchronous firing of a neuronal population can generate a PS potential in the extracellular recording at the pyramidal layer with neuronal somata, either a PS induced by a stimulation pulse or a PS generated spontaneously during an epileptic state. One of the recording channels, locating in the pyramidal layer of CA1 region and gaining the largest evoked PS, was used to extract PS waveforms. The stimulation artifacts in the recording signals were removed by a custom‐made MATLAB program. The program utilized an algorithm to detect and to replace each artifact segment (~ 1.0 ms) by an interpolation line connecting the two ends of the artifact segment.^33^

PS waveforms in the signals were then detected by a sliding time window (5 ms) with a threshold of minimum PS amplitude 0.5 mV. The PS rate was defined as the number of PS per second. The amplitude and width of PS waveform reflect the number of firing neurons and the degree of the firing synchronization. More firing neurons and higher degree of firing synchronization can both generate a larger amplitude and a shorter width of the PS. The area of PS waveform is proportional to the number of action potential units, that is, the number of firing neurons.^27^, ^34^ For clarity, the calculations of these PS indexes were illustrated in corresponding figures to accompany statistical data.

The histogram of the probability of inter‐spike intervals (ISI) of PSs was calculated with a resolution of 2 ms and a range of 0 ‐ 100 ms The accumulative ISI probabilities in the ranges of ISI < 8 ms and ISI = 9‐12 ms were used to evaluate the differences of PS features between the 60‐s and 5‐s HFS groups.

All the statistical data were presented as mean ± standard deviation, with "*n*" representing the number of rat experiments. The results of Shapiro‐Wilk test showed that the distributions of all the statistical data were normal. The comparisons of statistical data among groups with different HFS durations were analyzed using one‐way ANOVA or MANOVA with *Post hoc* Bonferroni tests, and comparisons between paired groups were analyzed by paired *t* test.

## 3 RESULTS

### 3.1 Afterdischarges induced by short HFS trains on afferent fiber in the CA1 region

A short HFS train (1, 3, 5, or 10 s) applied in the Schaffer collateral of CA1 region induced an afterdischarge (AD) with large PS events following the termination of HFS (Figure 1C). At the beginning of the HFS trains with different durations, the neuronal responses to the initial pulses were similar with a large first PS evoked by the very first pulse and attenuated PSs afterward (Figure 1C expanded insets at the right). Following the end of HFS, the length of AD varied with HFS durations. The mean AD duration induced by a 5‐s HFS was significantly longer than those induced by an HFS of 1, 3, or 10 s (Figure 1D). The mean PS rate within an AD induced by a 5‐s HFS was also maximum and significantly greater than 1‐ or 10‐s HFS (Figure 1E).

The results indicate that the short HFS trains can induce epileptiform activity (ie, AD) after the withdrawal of stimulation in the hippocampal CA1 region. Thus, we next utilized the epileptic AD model induced by a 5‐s short HFS to investigate the suppression effect of sustained HFS on the epileptic discharges.

### 3.2 Sustained HFS suppressed the epileptiform PS of afterdischarges

**Figure 2:**
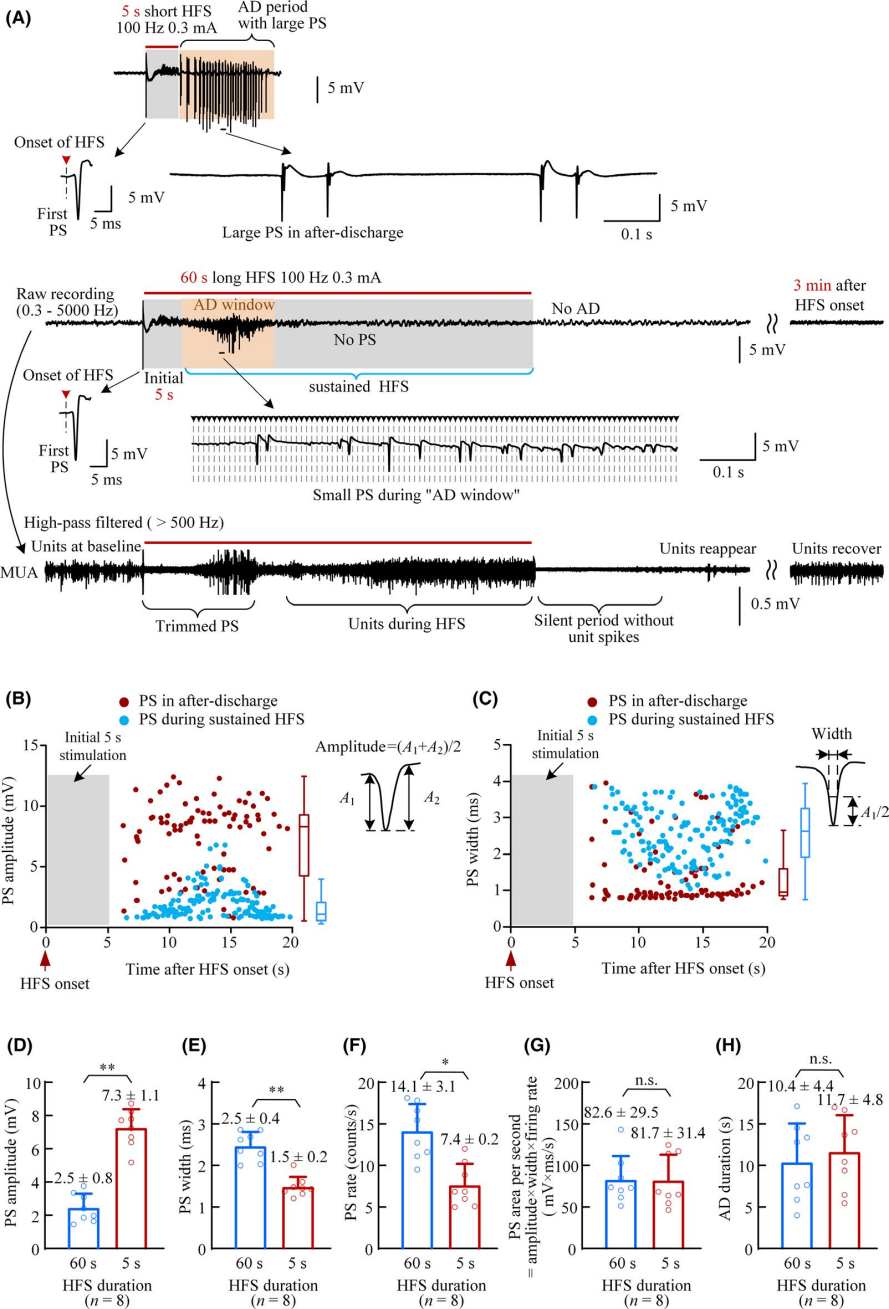
Sustained HFS suppressing epileptiform activity. (A) Examples of neuronal responses to 5‐s and 60‐s HFS at the Schaffer collaterals in the hippocampal CA1 region. The signal of multiple unit activity (MUA) obtained by high‐pass filtering the original recording shows a clear silent period following the termination of 60‐s HFS. (B) and (C) Scatter diagrams of the amplitudes and widths of the PS waveforms in the afterdischarges (AD) induced by the 5‐s short HFS and in the corresponding “AD window” induced by the sustained HFS shown in A. (D)–(H) Comparisons of the mean amplitude, width, rate of PS, PS area per second, and AD duration in the afterdischarges induced by 5‐s HFS and in the corresponding “AD window” of sustained HFS (**P < .01, *P < .05, paired *t* test, *n* = 8 rats). “n.s.” represents “no significant”

We prolonged the HFS duration immediately following the initial 5 s to 60 s to check whether a sustained stimulation could suppress an afterdischarge. For the paired trains of a short 5‐s and a long 60‐s HFS performed in a same rat preparation (Figure 2A), the neuronal responses in the initial 5‐s stimulations were similar. We termed the time window in 60‐s HFS corresponding to the AD period following the paired 5‐s HFS as “AD window.” With the sustained stimulation in the “AD window,” relatively small and wide PSs appeared rather than the large and narrow PSs in a real AD after the 5‐s stimulation (Figure 2B,C). Following the “AD window” period, during the remaining stimulation period of 60‐s HFS, no obvious PS appeared. In addition, no AD appeared following the end of 60‐s HFS (Figure 2A *the third row*). Instead, a silent period without unit spikes appeared immediately following the termination of 60‐s long HFS (Figure 2A *bottom*).

The statistical data showed that with similar amplitudes of initial PS evoked by the first pulse of HFS (5‐s HFS: 8.12 ± 3.98 mV, 60‐s HFS: 8.15 ± 3.45 mV, paired *t* test, *P* = .45, *n* = 8 rats), the mean amplitude of PSs during “AD window” within 60‐s HFS was only ~ 1/3 amplitude of PSs during real AD following 5‐s HFS (Figure 2D). The mean PS width and PS rate during “AD window” of 60‐s HFS were significantly greater than those of AD following 5‐s short HFS (Figure 2E,F). However, there was no significant difference in the mean PS areas per second between 60‐s and 5‐s HFS groups (Figure 2G), indicating similar amount of neuronal firing. In addition, measuring from the end of initial 5‐s, the PS periods lasted 10.4 ± 4.4 s in the 60‐s HFS, which was not significantly different from the length of AD following the 5‐s short HFS (11.7 ± 4.8 s, *n* = 8 rats, Figure 2H).

With a similar firing amount, the decrease of PS amplitude and the increase of PS width and PS rate suggested that the sustained HFS might disperse neuronal firing.

**Figure 3:**
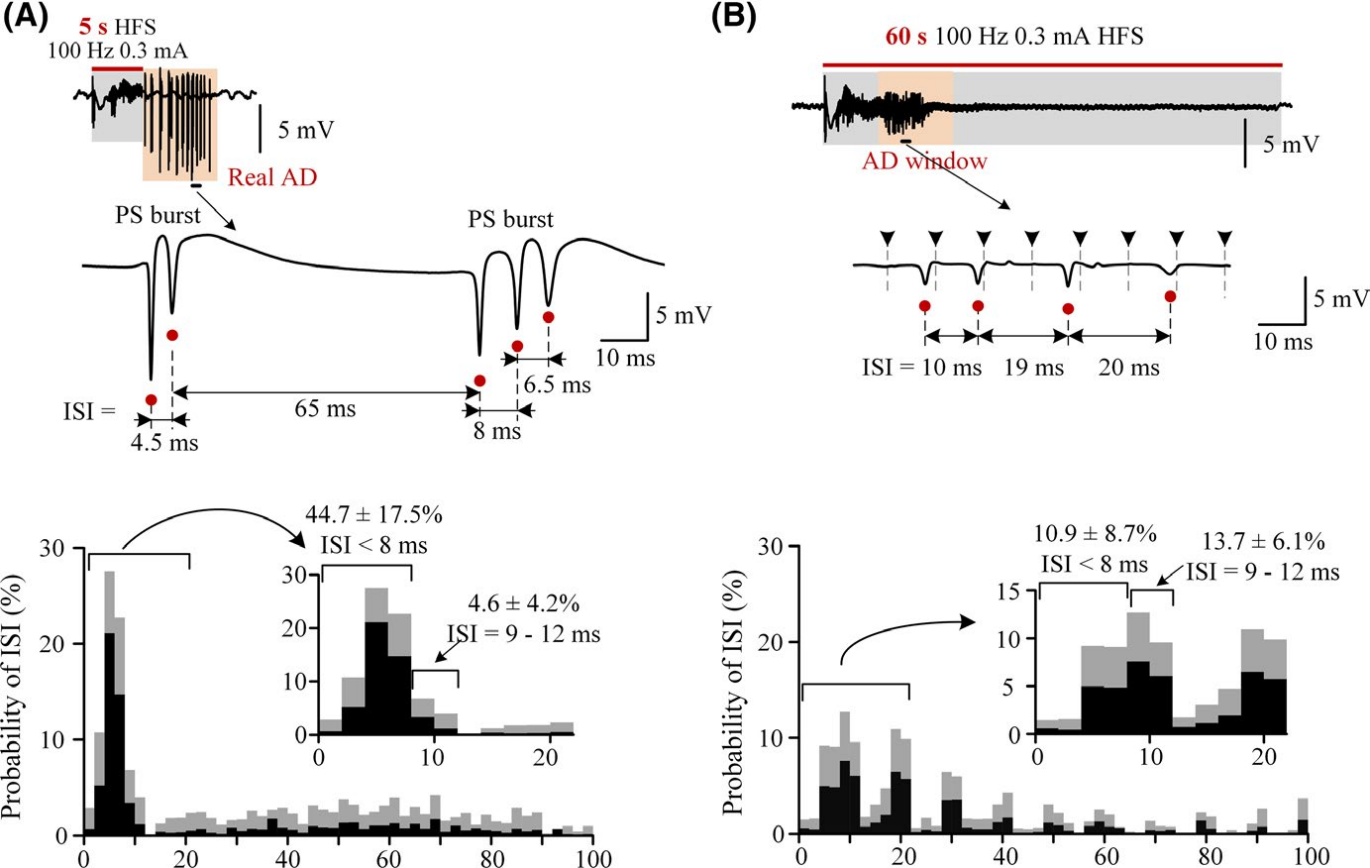
Sustained HFS altering the firing pattern of population spikes in the CA1 region. (A) *Top*: typical example of bursty PSs during an afterdischarge. Red dots denote PSs. *Bottom*: the average ISI histograms of PSs during afterdischarge period (*n* = 8 rats). The gray shade in the ISI histogram denotes the range of one standard deviation. An expanded insert in the ISI range 0 ‐ 22 ms is shown with the accumulative probabilities in the ranges of ISI < 8 ms and ISI = 9 ‐ 12 ms. (B) *Top*: example of PS events appeared in "AD window" during sustained HFS. *Bottom*: the average ISI histogram of PSs during "AD window" (*n* = 8 rats)

The distributions of ISI further showed the dispersing effect of sustained HFS (Figure 3). In ADs induced by the 5‐s short HFS, the large PS events appeared as bursts consisting of several successive PSs separated by ISIs shorter than ~ 8 ms which resulted in a great peak in the ISI histogram (Figure 3A), indicating “synchronous burst firing” of neuronal populations.^35^ The PS bursts may be caused by the bursty characteristic of pyramidal cells in the hippocampal region.^36^, ^37^ However, in the corresponding “AD window” of the 60‐s HFS, the small PS events appeared with more uniform ISI rather than bursts (Figure 3B).

Although the mean total number of PSs (141 ± 67) during a “AD window” of 60‐s HFS was significantly greater than the corresponding PS number (86 ± 37) in a real AD following a 5‐s short HFS (paired *t* test, *P* < .01, *n* = 8 rats), the mean probability of PS appearance within ISI range 0 ‐ 8 ms was only 10.9 ± 8.7% in a “AD window” of 60‐s HFS, significantly smaller than 44.7 ± 17.5% in a real AD of 5‐s HFS (Figure 3, paired *t* test, *P* < .01, *n* = 8 rats). In addition, during 60‐s HFS, small peaks appeared in the ISI histogram at multiples of the inter‐pulse interval (IPI) of HFS (ie, 10 ms for the 100 Hz HFS), indicating that most of the PSs were phase‐locked with the stimulation pulses of sustained HFS (Figure 3B), although not every pulse successfully evoked a PS event. The cumulative probability of PS appearance in the ISI range 9 ‐ 12 ms (around 10 ms) was only 13.7 ± 6.1% (*n* = 8 rats), indicating a few PSs evoked by two adjacent pulses (Figure 3B *bottom*).

These results indicate that sustained HFS can suppress large PS events that would otherwise have appeared in ADs without continuous stimulation. In addition, after the end of 60‐s long HFS, no epileptic AD appeared, indicating that the subsequent stimulation of long HFS might reverse the effect of initial stimulation. The generation of AD by a short HFS train is considered to be caused by the impairment of the effects of inhibitory synapses on the principal neurons in the CA1 region.^21^, ^38^ Therefore, we hypothesize that the sustained HFS might recover the inhibitory effects. To verify this hypothesis, we utilized a paired‐pulse test to evaluate the alterations of inhibitory effects in the CA1 region induced by a 5‐s short HFS and a 60‐s long HFS.

### 3.3 Sustained HFS accelerating the recovery of local inhibitions

**Figure 4:**
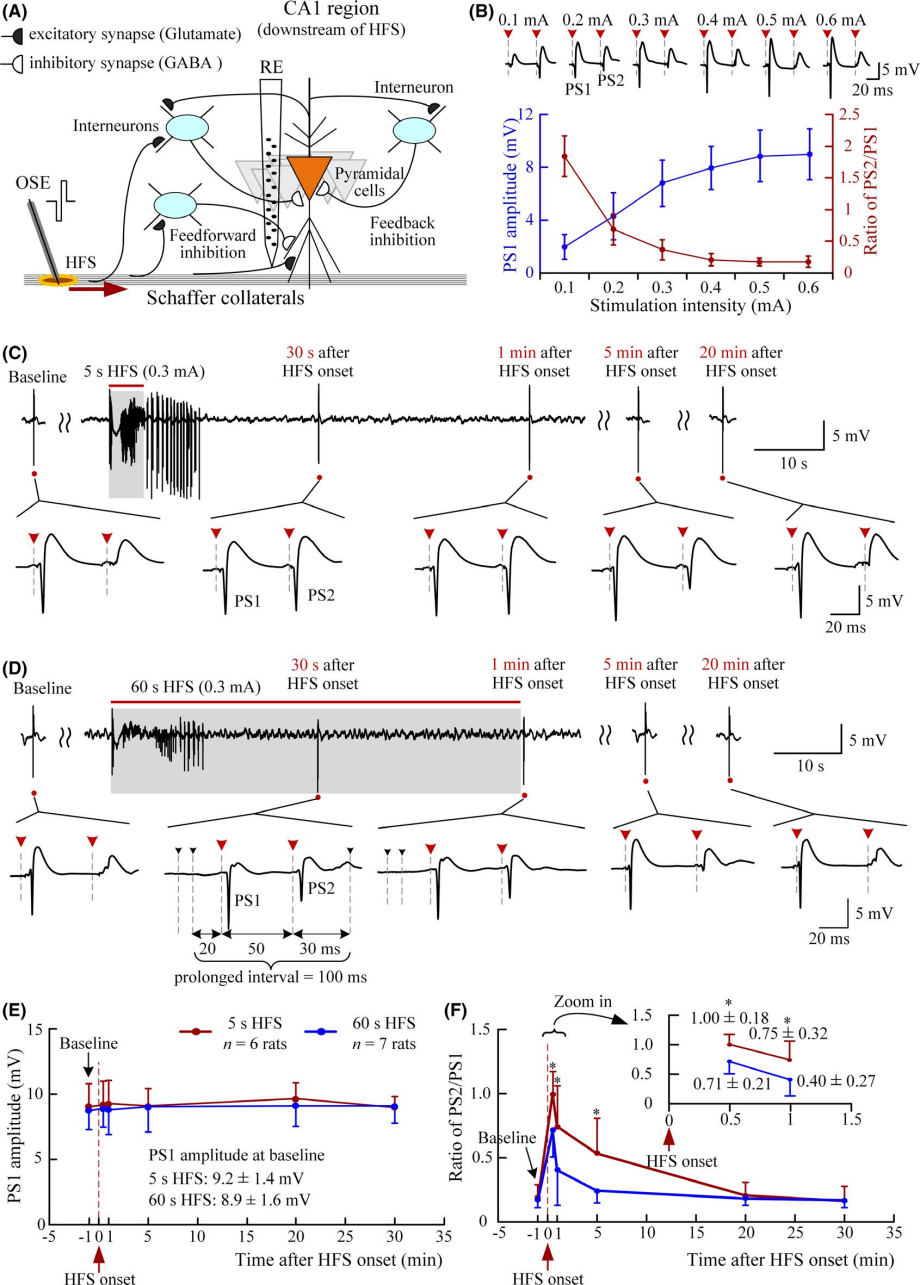
Sustained HFS accelerating the recovery of inhibition impairment induced by short HFS. (A) Schematic diagram of local inhibitory circuits in the hippocampal CA1 region with the electrodes. (B) Changes of the PS1 amplitudes and the PS2/PS1 ratios with the increase of stimulation intensity in paired‐pulse tests with an interval of 50 ms (*n* = 7 rats). Typical PS waveforms are shown above. (C) and (D) Typical examples of PS waveforms evoked by paired‐pulse tests before and after a 5‐s short HFS and before, during, and after a 60‐s HFS. (E) and (F) Comparisons of PS1 amplitude (control) and PS2/PS1 ratios between the two groups of 5‐s and 60‐s HFS at baseline and at the time of 30 s, 1, 5, 20, and 30 min after the onset of HFS (*P < .05, MANOVA with *post hoc* Bonferroni test)

The excitatory input from the Schaffer collaterals can activate both interneurons and pyramidal cells in the downstream CA1 region. The activated interneurons may inhibit pyramidal cells through the local circuit of feedforward inhibition; meanwhile, the activated pyramidal cells may inhibit themselves through the local circuit of feedback inhibition (Figure 4A).^37^, ^39^

These inhibitory effects can be measured by a paired‐pulse stimulation with a short interval (eg, 50 ms). With the increase of stimulus intensity from 0.1 to 0.6 mA, the amplitude of PS1 induced by the first pulse gradually increased, while after a 50 ms interval, the amplitude of PS2 induced by the second pulse decreased, thereby resulting a decrease of the PS2/PS1 ratio (Figure 4B). The suppression of PS2 is caused by both feedforward and feedback inhibitions and is termed as paired‐pulse depression (PPD).^40^

With the stimulus intensity (0.3 or 0.4 mA) used in the HFS trains, at baseline recording, the mean PS1 amplitude reached ~ 3/4 of maximal amplitude and the PS2/PS1 ratio was 0.17 ± 0.06 (*n* = 7 rats), indicating a strong inhibitory effect induced by the first pulse together with the PS1 (Figure 4B). The strong inhibition also appeared at the initial of 100 Hz HFS and indicated by the failure of several pulses to induce obvious PS after the first PS induced by the very first pulse (Figure 1C, the expended plot “a” on the *right*).

To investigate the change of inhibition caused by HFS trains, we evaluated the PPD by applying paired‐pulse tests (with a 50 ms interval) at the time of 30 s, 1, 5, 20, and 30 min after the onset of both 5‐s and 60‐s HFS trains. For a 5‐s HFS train, all the PPD tests were applied after the termination of AD thereby avoiding possible interferences of PS events of AD to the tests (Figure 4C). For a 60‐s HFS train, the PPD test at 30 s was in the middle of HFS. Therefore, a prolonged interval of 100 ms was inserted to allow the performance of a paired‐pulse test (Figure 4D).

Statistical data showed that the mean amplitudes of PS1 (the control) induced by PPD tests at the five test times for both the short and long HFS groups were all similar to the baseline levels and had no significant differences between the two groups (5‐s HFS vs. 60‐s HFS, MANOVA with *post hoc* Bonferroni test, *P* > .63, Figure 4E). In addition, at baseline recording, the mean PS2/PS1 ratios for the two HFS groups were small and similar (5‐s HFS: 0.19 ± 0.10, *n* = 6 rats; 60‐s HFS: 0.17 ± 0.06, *n* = 7 rats; MANOVA with *post hoc* Bonferroni test, *P* = .75). However, for the 5‐s HFS group, the mean PS2/PS1 ratios at 30 s, 1 min, and 5 min (1.00 ± 0.18, 0.75 ± 0.32 and 0.53 ± 0.27, *n* = 6 rats) were significantly greater than both the baseline level and the values of 60‐s HFS group at corresponding test times (0.71 ± 0.21, 0.40 ± 0.27 and 0.24 ± 0.09, *n* = 7 rats; MANOVA with *post hoc* Bonferroni test, *P* < .05; Figure 4F). For the 60‐s HFS group, only the mean PS2/PS1 ratios at 30 s and 1 min were significantly greater than the baseline level. They returned to the baseline level at 5 min earlier than the recovery of 5‐s HFS group (Figure 4F).

These results showed that with a similar control activation by the first pulse of PPD tests (indicated by a similar PS1 amplitude) and with a similar baseline inhibition, the impairment of PPD inhibition by a 5‐s HFS was greater than that by a 60‐s HFS.

## 4 DISCUSSION

The major finding of this study is that sustained HFS can significantly decrease the amplitude of epileptiform PSs and reduce PS bursts in the ADs induced by a short HFS, as well as generate a silent period instead of an AD following the termination of sustained HFS. The implication and possible mechanisms underlying the finding are discussed below.

### 4.1 Epileptic model for evaluating the effect of sustained HFS on neuronal firing

Sequences of short pulse stimulations with a duration of 1‐5 s and a pulse frequency around 100 Hz have been used to induce afterdischarges (AD) with seizure‐like activity in animals in vivo as well as in patients for clinical investigations in the brain regions such as hippocampus and cortex.^30^, ^41^, ^42^, ^43^ Previous studies have shown that the generation of AD is caused by the impairment and reversal of the inhibitory effects of GABAergic synapses on pyramidal cells in the hippocampal region.^44^, ^45^ A short HFS at axons may activate both pyramidal cells and interneurons in the projecting areas. Presumably, the interneurons would fire intensively under the impulses both from the HFS and from the firing of pyramidal cells and then activate GABAergic synapses on the pyramidal cells (Figure 4A). The excessive activation of the inhibitory synapses would cause an excessive influx of chloride ions and outflux of carbonate ions.^46^ The fast redistributions of ions in the two sides of the synapse membrane may result in an initial hyperpolarization followed by a late depolarization in the synaptic potentials,^45^, ^47^ thereby converting the GABAergic inhibition into excitation.^44^, ^48^ The conversion would destroy the balance between the inhibition and the excitation on the pyramidal cells thereby generating epileptic synchronous firing (ie, AD) of neuronal populations immediately following the cessation of a short HFS.^21^, ^45^

We utilized the AD model to evaluate the suppression effect of sustained HFS by using identical pulse parameters for both the 5‐s short HFS and the 60‐s long HFS. Since the long HFS actually contains the short HFS at its initial period, the 60‐s long HFS can be considered as a sustained 55‐s HFS applied immediately following a 5‐s short HFS. Therefore, the comparisons between the firing in the “AD window” of sustained HFS and the firing in the real AD after short HFS can provide direct evidence for the suppression effect of the subsequent stimulation. Our interesting finding is that an AD can appear with the absence of excitation from the subsequent stimulations, but may be suppressed by the subsequent stimulations. Therefore, the subsequent stimulation is no long epileptogenic but antiepileptic.

### 4.2 Desynchronization effect of stimulation may be an underlying mechanism for suppressing population spikes by sustained HFS

Previous studies have shown that prolonged HFS at axons may induce axonal conduction block, synaptic failure, and neurotransmitter depletion,^8^, ^49^, ^50^, ^51^ thereby attenuating the impulses to postsynaptic neurons. The attenuated effect of prolonged HFS may desynchronize the neuronal firing at the downstream CA1 region through a possible mechanism of intermittent activation of individual neurons by the HFS pulses.^22^, ^23^, ^52^ In addition, the block effects may prevent the propagation of epileptic activity from the upstream CA3 region to the downstream CA1 region, in case that the short HFS at the Schaffer collaterals may orthodromically and antidromically generate AD events in both the CA1 and CA3 regions, respectively. Thus, the blocking and dispersing effects may result in the suppression of population spikes by sustained HFS.

In addition, the results of paired‐pulse tests in the present study suggest another possible mechanism under the suppression effect of the sustained HFS. That is, the sustained HFS may accelerate the recovery of the impairment of GABAergic inhibition. Previous studies have shown that paired‐pulse depression (PPD) is caused by the strong inhibitions of the local inhibitory circuits in the CA1 region to suppress the second PS in a paired‐pulse stimulation.^53^, ^54^ The elimination of PPD following a 5‐s short HFS indicated a dysfunction of the inhibition while the restoration of PPD during sustained HFS suggested a recovery of the inhibition (Figure 4). In addition, the bursts of population spikes appeared during AD also indicate the impairment of the GABAergic inhibitions just as those induced by GABAergic antagonist.^55^, ^56^ Therefore, the alteration of PS bursts into separated PS by the sustained HFS may indicate a recovery of GABAergic inhibition, just as the separated PS induced by the very first pulse at the onset of HFS (or by a single pulse in baseline) when the GABAergic inhibition was intact (Figures 1C and 2A). Presumably, the asynchronous impulses of sustained HFS to the individual CA1 neurons dispersed the neuronal firing and stopped the conversion of GABAergic inhibition into excitation. The recovery of GABAergic inhibition would counterbalance the excitatory impulse of HFS pulses to prevent an increase of the total amount of neuronal firing during the “AD window” even with sustained HFS. Nevertheless, the PPD tests only provided indirect evidence of the inhibition recovery. Further studies are needed to obtain a direct demonstration.

Taken together, compared to the AD period with the absence of stimulation, during sustained HFS, both the de‐synchronous activation of the HFS pulses and the recovery of inhibitions may disperse neuronal firing without a significant change in the total amount of neuronal firing.

### 4.3 Implication and limitation

Highly synchronized firing of large population of neurons, such as the epileptic firing of large PS events during afterdischarges, may propagate to downstream regions through axonal projections and spread epileptiform activity widely in the brain. The suppression of PS events by sustained HFS can attenuate the strength of epileptic firing and prevent its propagation. Therefore, the effect of sustained HFS may be efficacious for treating certain types of epilepsy.

In addition, the study shows distinct effects of short HFS and sustained HFS. A short HFS can induce afterdischarges with large PS, indicating an epileptogenic effect. However, the sustained HFS can suppress synchronous firing, indicating an antiepileptic effect. Considering that some of DBS therapy in treating epilepsy may continue a stimulation of HFS for hours and days without pause,^4^, ^30^ for the paroxysmal activity of epileptic seizures, the ongoing HFS may look like beginning ahead and exert the antiepileptic effects of sustained HFS.

Moreover, axons occupy more space than other neuronal elements in brain and have a lower rheobase current.^17^ They are also more prone to be activated by the narrow pulses of HFS.^7^ Additionally, axons can widely modulate neuronal activity in downstream regions through their fiber projections.^3^, ^57^ Therefore, the results of axonal HFS in the hippocampal region in the present study should have a general significance in brain stimulation. Nevertheless, further studies are needed to investigate the suppression effects of HFS on epileptiform activity in other brain regions. Besides the stimulation in brain, stimulations in peripheral nerves, such as the vagus nerve stimulation (VNS), have also been investigated to treat drug‐resistant epilepsy.^58^ Our finding of axonal HFS may also provide clues for the investigations of VNS mechanisms.

A limitation of the present study is that the experiments were performed on urethane anesthetized rats. However, the urethane is a commonly used anesthetic in neuro‐electrophysiological experiments. It only has a slight suppression on neuronal excitability and neurotransmission.^59^ The large amplitudes ~ 8 mV of the initial PS orthodromically evoked by the first pulse of HFS and the large PSs during ADs (Figures 1, 2, 3, 4) also indicate an insignificant effect of the anesthetic on the neuronal activity in brain. Presumably, the effect of sustained HFS on epileptiform activity may also exert in an awake brain. Nevertheless, further studies with freely moving animals are needed to confirm the conclusion.

In addition, we only utilized the afterdischarge as an epileptic model to test the efficacy of sustained HFS. In fact, previous studies have reported that minutes‐long HFS has certain antiepileptic effects and can relieve the behavioral symptoms of seizures in the models of pentylenetetrazole or kainic acid induced seizure in rats to some extent.^60^, ^61^, ^62^ However, the underlying mechanisms are unclear. Our present study with the afterdischarge model and the direct comparisons between short and long HFS provides a possible mechanism of desynchronization effect of HFS. Other mechanisms, such as enhancing neuroprotection through glial cells and preventing neuron loss caused by seizures, may also underlie the effects of sustained HFS.^63^, ^64^, ^65^ With the great variety of pathological mechanisms of epilepsy, every treatment has its limitations. Studies on more epileptic models are needed to test the effects of HFS on other types of epilepsy and to reveal more mechanisms.

## 5 CONCLUSION

The present study shows that sustained HFS can suppress the highly synchronized firing in the hippocampal region by dispersing the firing of individual neurons without significantly altering the firing amount. The finding provides novel clues for treating epilepsy by high‐frequency pulses in DBS.

## CONFLICT OF INTEREST

The authors declare that they have no conflicts of interest.

## Data Availability

The data that support the findings of this study are available from the corresponding author upon reasonable request.
